# Altered dynamic neural activities in individuals with obsessive-compulsive disorder and comorbid depressive symptoms

**DOI:** 10.3389/fpsyt.2024.1403933

**Published:** 2024-08-08

**Authors:** Tinghuizi Shang, Yunhui Chen, Zhenning Ding, Weiqi Qin, Shancong Li, Siyi Wei, Zhipeng Ding, Xu Yang, Jiale Qi, Xiaoqing Qin, Dan Lv, Tong Li, Zan Pan, Chuang Zhan, Jian Xiao, Zhenghai Sun, Na Wang, Zengyan Yu, Chengchong Li, Ping Li

**Affiliations:** ^1^ Department of Psychiatry, Qiqihar Medical University, Qiqihar, Heilongjiang, China; ^2^ Medical Imaging Department, Qingdao Mental Health Center, Qingdao, Shandong, China; ^3^ The Second Affiliated Hospital, Qiqihar Medical University, Qiqihar, Heilongjiang, China; ^4^ Medical Technology Department, Qiqihar Medical University, Qiqihar, Heilongjiang, China; ^5^ Infection Control Department, Harbin Puning Hospital, Harbin, Heilongjiang, China; ^6^ Department of Psychiatry, Baiyupao Psychiatric Hospital of Harbin, Harbin, Heilongjiang, China

**Keywords:** obsessive-compulsive disorder, depressive symptoms, dynamic amplitude of low frequency fluctuation, magnetic resonance imaging, resting state

## Abstract

**Objectives:**

Depressive symptoms are the most prevalent comorbidity in individuals with obsessive-compulsive disorder (OCD). The objective of this study was to investigate the dynamic characteristics of resting-state neural activities in OCD patients with depressive symptoms.

**Methods:**

We recruited 29 OCD patients with depressive symptoms, 21 OCD patients without depressive symptoms, and 27 healthy controls, and collected data via structural and functional magnetic resonance imaging (fMRI). We analyzed the fMRI results using the dynamic amplitude of low-frequency fluctuation (dALFF) and support vector machine (SVM) techniques.

**Results:**

Compared with OCD patients without depressive symptoms, OCD patients with depressive symptoms exhibited an increased dALFF in the left precuneus and decreased dALFF in the right medial frontal gyrus. The SVM indicated that the integration of aberrant dALFF values in the left precuneus and right medial frontal gyrus led to an overall accuracy of 80%, a sensitivity of 79%, and a specificity of 100% in detecting depressive symptoms among OCD patients.

**Conclusion:**

Therefore, our study reveals that OCD patients with depressive symptoms display neural activities with unique dynamic characteristics in the resting state. Accordingly, abnormal dALFF values in the left precuneus and right medial frontal gyrus could be used to identify depressive symptoms in OCD patients.

## Introduction

Although obsessive-compulsive disorder (OCD) is one of the most common mental disorders, it is difficult to treat, and has a lifetime prevalence of 2–3% (1). The main clinical manifestations are intrusive thoughts (obsessions) and ritualistic behaviors (compulsions), and these have a high rate of coexistence with other psychiatric symptoms (2, 3). The presence of comorbid conditions influences the development, treatment response, and prognosis of OCD, and increases the risk of relapse (3). Statistically, depressive symptoms are the most common comorbidity, and around 50% of patients with OCD have a lifetime comorbidity of major depressive disorder (MDD) (4, 5). Previous studies have shown that obsessive-compulsive symptoms and functional impairment are more severe in OCD patients with depressive symptoms compared with those without depressive symptoms (6). Additionally, OCD patients with depressive symptoms are less likely to progress in treatment, have a prolonged illness duration, and are at greater risk of suicide (5, 7). Therefore, additional research is urgently needed to improve our understanding of the neural mechanisms of depressive symptoms in OCD patients.

Resting-state functional magnetic resonance imaging (rs-fMRI) is a popular method for investigating natural and spontaneous neural activity (8, 9). Based on blood-oxygen-level-dependent (BOLD) fMRI signals, we have used regional homogeneity, functional connectivity (FC) and dynamic FC methods to explore the characteristics of spontaneous neural activity in OCD patients at rest (10–12). The amplitude of low-frequency fluctuation (ALFF) is a viable and accurate metric for assessing local intrinsic brain activity (13, 14). Additionally, the sliding window technique can be used to capture the dynamic characteristics of brain activity in the temporal dimension (15, 16). The dynamic amplitude of low-frequency fluctuation (dALFF) is a combination of ALFF and sliding window technology. It can be used to quantify the dynamic attributes of neural activity in the brain by measuring the temporal variability of local neural activity between voxels (17, 18). As a result, dALFF is able to effectively capture the characteristics of temporal variations in intrinsic brain activity.

dALFF analysis has been used to investigate the neural mechanisms of various psychological conditions. For instance, patients with OCD had abnormal dALFF values in the cortical-striatal-thalamic-cortical (CSTC) pathway, bilateral inferior parietal lobule, and cerebellum (19). Additionally, abnormal dALFF values in the left cerebellum were negatively correlated with depressive symptoms in OCD patients (19). Meanwhile, in MDD patients, abnormal dALFF values were mainly found in the vermis, bilateral cerebellum posterior lobe, bilateral superior frontal gyrus, bilateral thalamus, and right middle frontal gyrus (20, 21). In these patients, depression severity was positively associated with elevated dALFF values in the right thalamus and right cerebellar posterior lobe (20). Abnormal temporal homogeneity of resting-state dynamic neural activity has been observed in patients with OCD and MDD (19, 20). Currently, whether individuals with OCD and depressive symptoms exhibit specific or distinct changes in dynamic neural activity at rest remains uncertain. These abnormalities could serve as diagnostic markers of OCD with depressive symptoms, warranting further examination.

Support vector machine (SVM) analysis is a form of supervised machine learning in which generalized linear classifiers are used to conduct binary data categorization (22). SVM analysis provides optimal classification by building a super-optimal layer from a good data set (23). Because of its specificity and validity, SVM analysis has been broadly applied when making predictions regarding neuroimaging datasets (24). Consequently, we employed the SVM technique in the present study to ascertain whether abnormal resting-state dynamic neural activity can be used to discriminate depressive symptoms in OCD patients.

In this study, we used both dALFF and SVM analysis to investigate the dynamic characteristics of spontaneous brain activity in the resting state in OCD patients with depressive symptoms. We hypothesized that OCD patients with depressive symptoms might exhibit abnormal dALFF values in certain brain areas, and that these could be used to identify depressive symptoms in OCD patients.

## Materials and methods

### Participants

We recruited 50 OCD patients and 27 age- and education-matched healthy controls (HCs). Two psychiatrists diagnosed the OCD patients according to the criteria of the 5th edition of the Diagnostic and Statistical Manual of Mental Disorders (DSM-5). OCD severity, anxious symptoms, and depressive symptoms in the OCD patients were assessed using the Yale-Brown Obsessive Compulsive Scale (Y-BOCS), the Hamilton Anxiety Rating Scale (HAMA), and the 17-item Hamilton Rating Scale for Depression (HAMD_17_), respectively. Only patients with a Y-BOCS total score of 16 or above and a HAMD_17_ score below 18 were included in the study. Based on HAMD_17_ scores, the OCD patients were categorized into two subsets. There were 29 OCD patients with depressive symptoms (HAMD_17_ score ≥ 8) and 21 OCD patients without depressive symptoms (HAMD_17_ score ≤ 7) (25, 26). In this study, the OCD patients with depressive symptoms did not meet the diagnostic criteria for comorbid MDD. Participants were not included if they met the following exclusion criteria (1): severe physical disease or neurological disorders; (2) past or present drug or alcohol abuse; (3) women who were pregnant or lactating; and (4) individuals with contraindications for MRI. In addition, each participant was ethnically Han Chinese, right-handed, and aged 18–45 years.

This study was approved by the Medical Ethics Committee of Qiqihar Medical University. Informed consent was gained from each subject before study enrolment.

### Image acquisition and preprocessing

All subjects underwent rs-fMRI scanning on a 3.0 T MRI scanner. rs-fMRI scans were captured using an echo planar imaging sequence with the following specifications: A repetition time (TR) of 2000 ms, echo time (TE) of 30 ms, flip angle of 90°, field of view (FOV) measuring 220 × 220 mm, matrix size of 64 × 64, slice thickness of 4 mm, no gap, 32 slices, and a total of 240 volumes.

The first 10 volumes were eliminated to account for signal equilibration. The remaining fMRI volumes were pre-possessed using SPM8 software, including steps for slice timing correction, realignment, spatial normalization to MNI space using an EPI template, and smoothing with a 6-mm Gaussian kernel. Bandpass filtering (0.01–0.08 Hz) and linear detrending were used to minimize high-frequency physiological noise and low-frequency drifts, respectively.

### dALFF analysis

dALFF analysis was performed using REST plus-based temporal dynamic analysis toolkits (27). We used a sliding-window approach to delineate dALFF throughout the whole brain. According to previous studies (20, 28, 29), we used a window length of 50 TRs and a step size of 1 TR to compute the dALFF for each individual. The time series data from each individual was segmented into 181 windows, and an ALFF map was calculated for each window.

### Statistical analysis

Statistical analyses were conducted using SPSS 22.0 for both the clinical and demographic data. Classification data were examined using the Chi-square test. First, we examined the homogeneity of variance and normal distribution of all continuous variables. We used one-way analyses of variance (ANOVA) or two-sample *t*-tests to assess continuous variables with a normal distribution or homogeneity of variance. For other variables, we used the Kruskal-Wallis *H* test. A significance threshold of *P* < 0.05 (two-tailed) was established for all statistical tests conducted.

Second-level analyses were performed in SPM8 to compare differences in dALFF between the groups. Specifically, we sought to identify brain regions exhibiting significant variations in dALFF among the OCD patients with depressive symptoms, OCD patients without depressive symptoms, and HC groups. *Post-hoc* two-sample *t*-tests were performed to assess differences in dALFF values among various group combinations. To adjust for multiple comparisons, we applied Gaussian random field (GRF) theory with the voxel-level significance threshold set at *P* < 0.001 and the cluster-level threshold set at *P* < 0.05 (30, 31).

Associations between abnormal dALFF values and Y-BOCS, HAMD_17_, and HAMA scores were investigated using Pearson’s analysis and the Bonferroni correction for all patients, OCD patients with depressive symptoms, and OCD patients without depressive symptoms.

### SVM analysis

In this study, we used the linear SVM technique from the LIBSVM package in MATLAB (32) to identify OCD patients with depressive symptoms. This discrimination process was based on the differences in dALFF values for specific brain regions between groups. Considering the small sample size, we sought to avoid overfitting. Accordingly, the classifier was first tested using the “leave-one-out” cross-validation method, in which a single subject was excluded from each group, and the remaining subjects were used to train the classifier. The excluded subjects were then used to test the performance of the classifier, and to build a hyperplane that could optimally distinguish different categories (33). This process was repeated for each subject to obtain the overall sensitivity, accuracy, and specificity (34). A permutation test was used to verify the SVM results, and 5,000 displacement tests were performed on each sample to test the statistical significance of the classification accuracy (35). Finally, the performance of the SVM model was verified by calculating the receiver operating characteristic curve (ROC) and area under the curve (AUC).

## Results

### Demographic and clinical characteristics of the participants

There were no noteworthy disparities in terms of age, sex, education, or head movement parameters among the three groups. Additionally, the duration of illness was not statistically different between the two OCD groups. Significant differences were found among the three groups in terms of the Y-BOCS total score, obsessive subscale score, and compulsive subscale score, as well as the HAMD_17_ and HAMA scores (**Table 1**). The *post-hoc* tests indicated no significant differences in Y-BOCS total scores, obsessive subscale scores, and compulsive subscale scores between the two OCD groups. However, the HAMD_17_ and HAMA scores of the OCD patients with depressive symptoms were significantly higher than those of the OCD patients without depressive symptoms (**Supplementary Table S1**).

**Table 1:** Demographic and clinical characteristics of participants.

Variables	OCD with depressive symptoms (n=29)	OCD without depressive symptoms (n=21)	HCs (n=27)	χ^2^/*F*/*t*/*H*	*P*-value
Age (years)	25.55 ± 7.40	27.48 ± 8.77	25.78 ± 8.72	0.37a	0.69
Gender (male/female)	17/12	12/9	20/7	1.97^b^	0.37
Education (years)	12.90 ± 2.90	13.52 ± 2.98	12.19 ± 3.40	1.12^a^	0.33
Illness duration (months)	72.69 ± 71.79	52.10 ± 73.69	–	0.99^c^	0.33
Y-BOCS total score	26.38 ± 5.57	23.95±6.58	1.07 ± 0.92	53.43^d^	<0.001^*^
Obsessive subscale score	13.59 ± 4.32	12.29±4.20	0.41 ± 0.50	51.76^d^	<0.001^*^
Compulsive subscale score	12.79 ± 3.56	11.67±5.18	0.63 ± 0.74	45.73^d^	<0.001^*^
HAMD_17_	11.38 ± 3.16	4.76±2.34	1.26 ± 0.98	62.09^d^	<0.001^*^
HAMA	13.83 ± 7.47	7.24±4.07	1.33 ± 0.83	52.03^d^	<0.001^*^
FD(mm)	0.07±0.02	0.07±0.03	0.07±0.04	0.34^a^	0.71

Data was displayed with mean ± standard deviation. OCD, obsessive-compulsive disorder; HCs, healthy controls; Y-BOCS, Yale-Brown Obsessive-Compulsive Scale; HAMD_17_, 17-item Hamilton Depression Rating Scale; HAMA, Hamilton Anxiety Rating Scale; FD, framewise displacement.

^a^ANOVA.

^b^Pearson chi-square.

^c^Two sample t-test.

^d^Kruskal-Wallis H.

^*^ *P* < 0.001 when comparing variables between the three groups. The results of post-hoc comparisons among individual groups are shown in the **Supplementary Materials** (**Supplementary Table S1**).

### Differences in dALFF values among groups

There were no significant differences in the dALFF values among the three groups (voxel *p* < 0.001 and cluster *p* < 0.05 GRF corrected).

**Figure 1:**
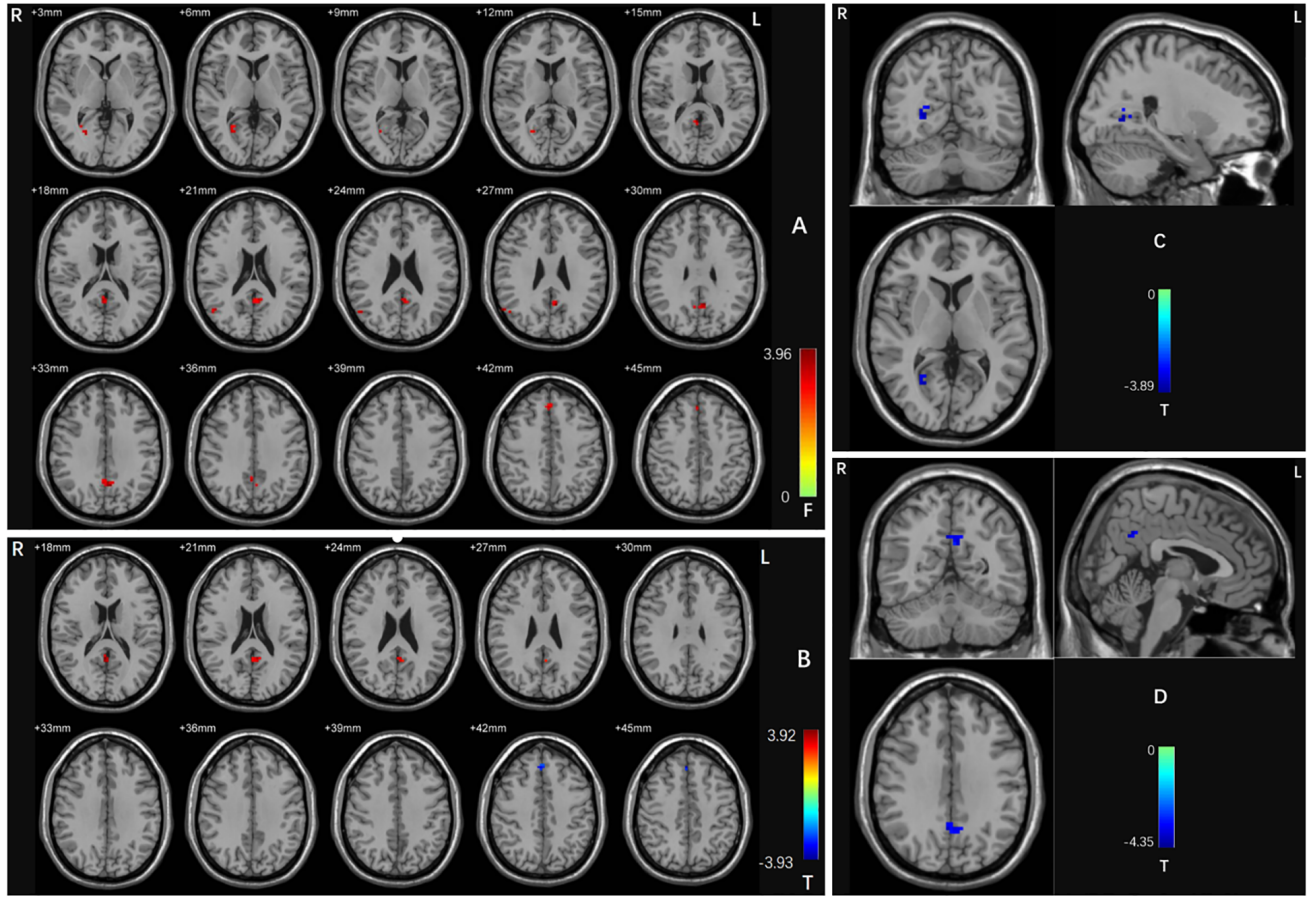
**(A)** Brain regions with abnormal dALFF values in the three groups based on an ANCOVA. Red shades denote significant differences among groups. The color bar indicates the *F* value from the ANCOVA. **(B)** Brain regions with abnormal dALFF values between the OCD patients with and without depressive symptoms based on *post-hoc t*-tests. Red shades denote high dALFF values and blue shades denote low dALFF values. The colored bars indicate the *T* value. **(C)** Brain regions with abnormal dALFF values between OCD patients with depressive symptoms and HCs based on *post hoc t*-tests. Blue shades denote low dALFF values. The colored bars indicate the *T* value. **(D)** Brain regions with abnormal dALFF values between OCD patients without depressive symptoms and HCs based on *post hoc t*-tests. Blue shades denote low dALFF values. The colored bars indicate the *T* value. OCD, obsessive-compulsive disorder; HCs, healthy controls.

A one-way ANOVA revealed significant differences in dALFF values across the three groups in four clusters: the right calcarine cortex, right middle temporal gyrus, left precuneus, and right medial frontal gyrus (voxel *p* < 0.005 and cluster *p* < 0.05 GRF corrected) (**Table 2**; **Figure 1A**).

**Table 2:** Brain regions with abnormal dALFF values in patients with OCD.

Cluster location	Peak (MNI)			Number of voxels	*F/T* value
	x	y	z		
ANOVA					
Right calcarine cortex	24	-63	3	12	3.399
Right middle temporal gyrus	60	-63	27	8	3.606
Left precuneus	-6	-57	27	42	3.960
Right medial frontal gyrus	3	36	45	9	3.300
OCD with depressive symptoms vs.OCD without depressive symptoms					
Left precuneus	3	-51	18	20	3.921
Right medial frontal gyrus	3	36	45	9	-3.939
OCD with depressive symptoms vs.HCs					
Right calcarine cortex	24	-63	6	12	-3.897
OCD without depressive symptoms vs.HCs					
Left precuneus	3	-57	33	30	-4.352

dALFF, dynamic amplitude of low-frequency fluctuation, OCD, obsessive-compulsive disorder; HCs, health controls; MNI, Montreal Neurological Institute.

Compared with the OCD patients without depressive symptoms, those with depressive symptoms had significantly increased dALFF values in the left precuneus and decreased dALFF values in the right medial frontal gyrus (voxel *p* < 0.005 and cluster *p* < 0.05 GRF corrected) (**Table 2**; **Figure 1B**).

When comparing the OCD patients with depressive symptoms with the HCs, we detected a decreased dALFF value in the right calcarine cortex (voxel *p* < 0.005 and cluster *p* < 0.05 GRF corrected) (**Table 2**; **Figure 1C**). When comparing the OCD patients without depressive symptoms with the HCs, we discovered a significantly reduced dALFF value in the left precuneus (voxel *p* < 0.005 and cluster *p* < 0.05 GRF corrected) (**Table 2**; **Figure 1D**).

### Correlations between dALFF and clinical characteristics

The clinical variables (including the Y-BOCS total and subscale scores, HAMA and HAMD_17_ scores, and illness duration) were not correlated with abnormal dALFF values in all patients grouped together, the OCD patients with depressive symptoms, or the OCD patients without depressive symptoms.

### SVM results

**Figure 2:**
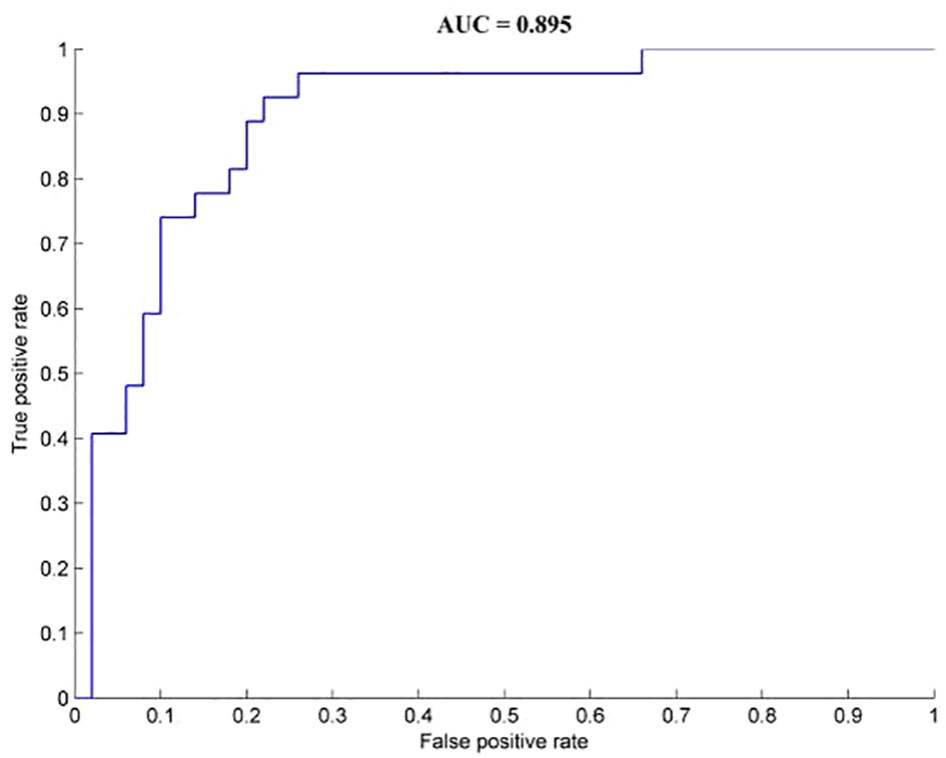
Classification performance for OCD and HCs combined with dALFF results. OCD, obsessive-compulsive disorder; HCs, healthy controls.

Brain regions with abnormal dALFF values among the three groups were used as feature variables in the classification models (1 = right calcarine, 2 = right middle temporal gyrus, 3 = left precuneus, 4 = right medial frontal gyrus). The integration of all of the feature variables led to the best discrimination of OCD patients from HCs, with precision, sensitivity, and specificity rates of 0.81, 0.93, and 0.78, respectively (**Figure 2**).

**Figure 3:**
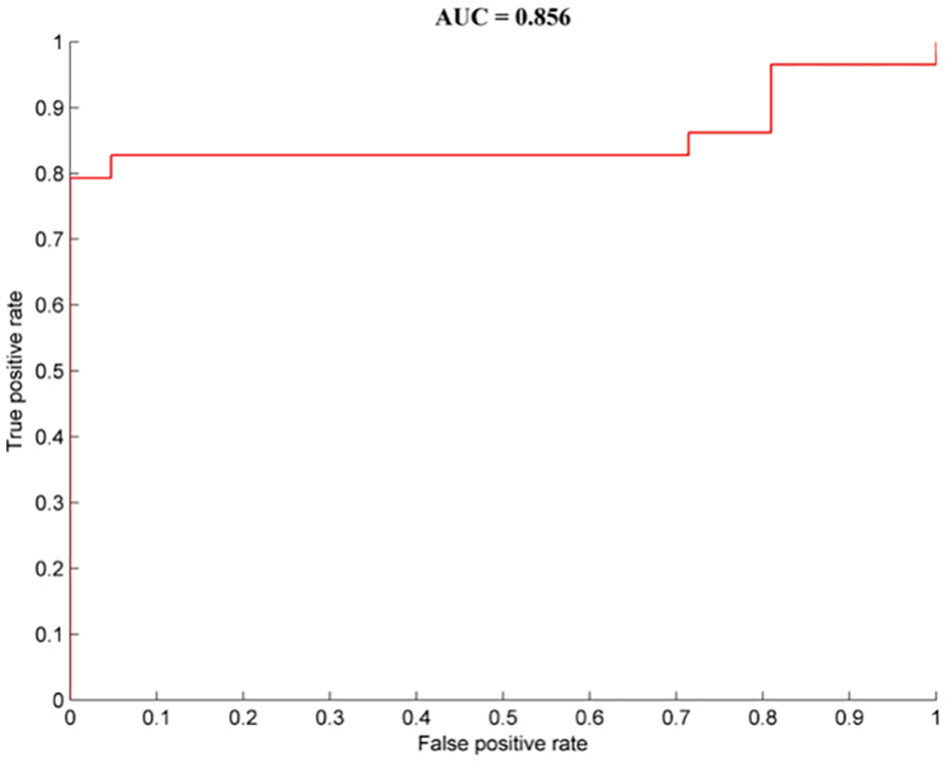
Classification performance for OCD patients with depressive symptoms from OCD patients without depressive symptoms combined with dALFF results. OCD, obsessive-compulsive disorder; HCs, healthy controls.

Brain regions with abnormal dALFF values between the OCD patients with depressive symptoms and those without depressive symptoms were represented as feature variables in the classification models (5 = left precuneus, 6 = right medial frontal gyrus). The combination of all of the feature variables could effectively distinguish OCD patients with depressive symptoms from those without depressive symptoms with precision, sensitivity, and specificity rates of 0.80, 0.79, and 1.00, respectively (**Figure 3**).

## Discussion

Our study revealed that OCD patients with depressive symptoms had more severe symptoms than those without depressive symptoms, as reflected by the higher HAMD_17_ and HAMA scores. In addition, OCD patients with depressive symptoms exhibited increased dALFF values in the left precuneus and reduced dALFF values in the right medial frontal gyrus compared with OCD patients without depressive symptoms. Moreover, the integration of changed dALFF values in the right medial frontal gyrus and left precuneus had good specificity and sensitivity for discriminating OCD patients with versus without depressive symptoms.

Previous studies have indicated that the severity of symptoms, especially those involving avoidance and obsessions, may be related to depression symptoms in OCD patients (36, 37). Additionally, OCD patients with depressive symptoms are more difficult to treat and have a longer course of disease (38, 39). Our study revealed that OCD patients with depressive symptoms had increased dALFF values in the left precuneus and reduced dALFF values in the right medial frontal gyrus when compared with OCD patients without depressive symptoms. Decreased gray matter volume and decreased ALFF values in the precuneus have been reported in OCD patients (40, 41). Previous studies have also reported that ALFF values in the precuneus in MDD patients were increased at rest and in an activated state, yet significantly decreased after treatment (42, 43). Given that abnormal precuneus activity may impair the regulation of negative emotions (44, 45), the increased dynamic neural activity in the left precuneus observed in the current study may contribute to depressive symptoms in OCD patients.

The medial frontal gyrus is involved in emotional expression and evaluation (46, 47). Emerging evidence suggests that structural and functional changes in the medial frontal gyrus are correlated with depressive symptoms (46, 48). A positron emission tomography study revealed that MDD patients had reduced metabolism in the medial frontal gyrus (49). In addition, decreased activation in the bilateral medial frontal gyrus has been found in the task-state in OCD patients, which may lead to a reduced ability to detect and eliminate unwanted obsessive thoughts and compulsive behaviors (50, 51). Therefore, decreased neural activity in the medial frontal gyrus may be a common pathogenesis of MDD and OCD.

The left precuneus and right medial frontal gyrus are critical components of the default-mode network (DMN), which is implicated in introspective and reflective self-awareness processes (52). DMN activity at rest was significantly elevated in patients with MDD (53) and decreased in patients with OCD (54). The abnormal dALFF values in the left precuneus and right medial frontal cortex found in the present study may represent altered dynamic DMN activity in OCD patients with depressive symptoms. This could be investigated in future research. Additionally, our SVM data revealed that the integration of elevated dALFF values in the left precuneus and reduced dALFF values in the right medial frontal gyrus led to higher accuracy in discriminating OCD patients with versus without depressive symptoms.

Compared with HCs, OCD patients with depressive symptoms had lower dALFF values in the right calcarine cortex. The gray matter volume in the calcarine cortex was increased in patients with late-onset depression (55), and the dALFF values in the calcarine cortex were reduced in MDD patients (56). In addition, reduced ALFF values and a lower degree of centrality have been reported in patients with OCD (57). Therefore, dynamic changes in neuronal activity in the calcarine cortex may be associated with the simultaneous occurrence of OCD and depressive symptoms.

Inconsistent with our hypothesis, abnormal dALFF values were not correlated with clinical parameters in all patients. Previous studies have also reported that changes in brain activities were not related to clinical parameters in patients with OCD (57, 58). Indeed, the altered dALFF values were heterogeneous among OCD patients, as previously reported (59, 60). In addition, the relatively small sample size and strict parameters for the Bonferroni correction may have influenced the current results (61, 62).

The current study has several limitations that should be noted. First, the optimal length of the activity window for obtaining dynamic neural activity data has not been established. Second, the statistical power of our analysis in terms of identifying changes in dynamic brain activity might have been constrained because of the small sample size in the current study. Third, as this was a cross-sectional study, we did not determine whether the abnormal dALFF values were a result or a cause of OCD with depressive symptoms. Fourth, in this study, the HAMA scores of the OCD patients with depressive symptoms were significantly higher than those of the OCD patients without depressive symptoms. Although the HAMA scores were not correlated with abnormal dALFF values, anxiety symptoms in the OCD patients might have impacted the obtained dALFF values. Therefore, we hope to verify the results of this study with purer samples in future work. Finally, since the voxel-level significance threshold was *p* < 0.005 for multiple comparisons by GRF corrected, the results of this study should be explained with caution.

## Conclusions

Our study revealed that OCD patients with depressive symptoms displayed unique dynamic neural activities at rest. Abnormal dALFF values in the left precuneus and right medial frontal gyrus may be useful in identifying OCD patients with depressive symptoms.

## Data availability statement

The original contributions presented in the study are included in the article/**Supplementary Material**. Further inquiries can be directed to the corresponding author.

## Ethics statement

The studies involving humans were approved by Medical Ethics Committee of Qiqihar Medical University. The studies were conducted in accordance with the local legislation and institutional requirements. The participants provided their written informed consent to participate in this study.

## Author contributions

TS: Formal analysis, Writing – original draft, Data curation. YC: Writing – original draft, Investigation, Methodology. ZND: Writing – original draft, Data curation. WQ: Investigation, Writing – original draft. SL: Investigation, Writing – original draft. SW: Investigation, Writing – original draft. ZPD: Formal analysis, Writing – original draft. XY: Investigation, Writing – original draft. JQ: Investigation, Writing – original draft. XQ: Investigation, Writing – original draft. DL: Investigation, Writing – original draft. TL: Investigation, Writing – original draft. ZP: Investigation, Writing – original draft. CZ: Methodology, Writing – review & editing. JX: Investigation, Writing – original draft. ZS: Investigation, Writing – original draft. NW: Investigation, Writing – original draft. ZY: Investigation, Writing – original draft. CL: Investigation, Writing – original draft. PL: Writing – review & editing, Project administration, Supervision.
