# Supplementary material for: Altered dynamic neural activities in individuals with obsessive-compulsive disorder and comorbid depressive symptoms
Source: Front Psychiatry. 2024 Aug 8;15:1403933. doi: 10.3389/fpsyt.2024.1403933 (PMC11339690; doi:10.3389/fpsyt.2024.1403933)
Supplement: Supplementary file 1 [file Table_1.pdf]

# *Supplementary Material*

**Table S1**

Table S1 The results of post-hoc tests comparing groups to one another for differential variables

| Variables | OCD with depressive symptoms vs. OCD without depressive symptoms (*P*-value) | OCD with depressive symptoms vs. HCs (*P*-value) | OCD without depressive symptoms vs. HCs (*P*-value) |
|---|---|---|---|
| Y-BOCS total score | 0.45 | $<$0.001 | $<$0.001 |
| Obsessive subscale score | 0.64 | $<$0.001 | $<$0.001 |
| Compulsive subscale score | 0.78 | $<$0.001 | $<$0.001 |
| $HAMD_{17}$ | $<$0.001 | $<$0.001 | $<$0.001 |
| HAMA | $<$0.001 | $<$0.001 | $<$0.001 |

OCD, obsessive-compulsive disorder; HCs, healthy controls; Y-BOCS, Yale-Brown Obsessive-Compulsive Scale; $HAMD_{17}$, 17-item Hamilton Depression Rating Scale; HAMA, Hamilton Anxiety Rating Scale.
